# Mitotic cell death induction by targeting the mitotic spindle with tubulin-inhibitory indole derivative molecules

**DOI:** 10.18632/oncotarget.14980

**Published:** 2017-02-01

**Authors:** Erica Di Cesare, Annalisa Verrico, Andrea Miele, Maria Giubettini, Paola Rovella, Antonio Coluccia, Valeria Famiglini, Giuseppe La Regina, Enrico Cundari, Romano Silvestri, Patrizia Lavia

**Affiliations:** ^1^ Institute of Molecular Biology and Pathology, CNR National Research Council, c/o Department of Biology and Biotechnology, Sapienza Università di Roma, Roma, Italy; ^2^ Present address: IRBM Science Park, Advent Srl, Pomezia, Italy; ^3^ Present address: EMBL, Heidelberg, Germany; ^4^ Istituto Pasteur Italia–Fondazione Cenci Bolognetti, Department of Drug Chemistry and Technology, Sapienza Università di Roma, Roma, Italy

**Keywords:** mitotic spindle microtubules, tubulin inhibitors, mitotic cell death, caspase-3, time-lapse imaging

## Abstract

Tubulin-targeting molecules are widely used cancer therapeutic agents. They inhibit microtubule-based structures, including the mitotic spindle, ultimately preventing cell division. The final fates of microtubule-inhibited cells are however often heterogeneous and difficult to predict. While recent work has provided insight into the cell response to inhibitors of microtubule dynamics (taxanes), the cell response to tubulin polymerization inhibitors remains less well characterized. Arylthioindoles (ATIs) are recently developed tubulin inhibitors. We previously identified ATI members that effectively inhibit tubulin polymerization *in vitro* and cancer cell growth in bulk cell viability assays. Here we characterise in depth the response of cancer cell lines to five selected ATIs. We find that all ATIs arrest mitotic progression, yet subsequently yield distinct cell fate profiles in time-lapse recording assays, indicating that molecules endowed with similar tubulin polymerization inhibitory activity *in vitro* can in fact display differential efficacy in living cells. Individual ATIs induce cytological phenotypes of increasing severity in terms of damage to the mitotic apparatus. That differentially triggers MCL-1 down-regulation and caspase-3 activation, and underlies the terminal fate of treated cells. Collectively, these results contribute to define the cell response to tubulin inhibitors and pinpoint potentially valuable molecules that can increase the molecular diversity of tubulin-targeting agents.

## INTRODUCTION

Microtubules (MTs) are tubulin polymers indispensable for a variety of cellular functions, including cell morphology and motility, intracellular transport, and mitotic spindle assembly and dynamic activity. Mitotic spindle impairment arrests cells in early mitotic stages and can lead them to death. MTs are therefore among the best-validated targets in cancer therapy. MT-targeting agents have empirical therapeutic efficacy in a variety of cancers [see 1-6 for reviews]. Targeting non-tubulin mitotic factors (e.g., mitotic kinases, kinesins, motor proteins and other mitotic regulatory factors) also reduces cell proliferation and increases the death rate of cancer cells [7–10]. These approaches indicate that mitosis remains a valuable therapeutic target, encouraging efforts to rationalize the design of mitosis-targeting molecules and to understand the molecular bases of the cell response to these molecules [11].

Although a variety of tumor types are sensitive to MT-targeting agents, there are limitations to their therapeutic use. First, the expression profile of genes encoding MT-associated and MT-regulatory proteins found in different cancer types, and also within cells from a same cancer, can synergize with, or antagonize, the effect of the drugs [12–14]. Second, tumor cells can develop resistance through diverse mechanisms [4, 15–17], involving not only the induction of efflux pump mechanisms implicated in multidrug resistance, but also the presence of mutations or differential expression of tubulin isotypes [18–21]. There is therefore a need to increase the molecular repertoire of potentially effective drugs.

Tubulin-targeting agents can either prevent MT polymerization or block their dynamic functions [1, 22]. In both cases dividing cells arrest in early mitosis with a defective mitotic apparatus and can eventually undergo death in mitosis, a molecularly distinct response from other cell death pathways [8, 23–25]. Cells treated with MT-targeting agents show variable propensity to undergo mitotic cell death, even among cells from genetically homogeneous cell populations [26–27]. Recent studies have sought to identify the determinants of mitotic death induction using *in vivo* imaging methods to follow up mitotic cell fates at the single cell level. In the case of Taxol (TAX), the prototype MT-stabilizing drug, a framework defined the “competitive networks” model has been formalized [28–30]. The model proposes that the heterogeneity in cell fates reflects the timing with which mitotic MT activity is blocked in individual cells: different cells would be endowed with a specific balance of pro-death and pro-mitotic factors, depending on their stage when TAX “hits” them. The model predicts that TAX-dependent block of MT dynamics in cells with abundant cyclin B1 determines sustained mitotic arrest, allowing time for the accumulation of pro-death factors that eventually induce cell death. Below a critical cyclin B1 threshold, instead, mitotic arrest would not be sustained for long enough to accumulate death factors, facilitating mitotic slippage with unsegregated or mis-segregating chromosomes. c-Myc, BH3-only proteins and BCL-xL play critical roles in the final fate of TAX-treated cells [30]. In the case of drugs that inhibit MT assembly, the links between the mechanisms of MT inhibition, the induction of mitotic arrest and the onset of mitotic death remain less well clarified.

In the last years, various strategies have been employed to design diversified molecules and expand the molecular repertoire of anti-mitotic compounds. We previously reported the development of a novel class of MT-targeting agents based on an arylthioindole scaffold (ATI), which was designed to bind selectively the Colchicine-binding site on tubulin. Colchicine binding inhibitors are regarded as promising compounds in recent studies [31–32]. Structure-activity relationship (SAR) and biological studies of ATIs [33–36] pinpointed potentially interesting features: i) they competitively inhibit the binding of [^3^H]Colchicine to tubulin *in vitro*; ii) they show good chemical and metabolic stability in microsomal fractions from the human and mouse liver, as well as in whole injected mice [33], iii) they are small molecules (<400 kDa) compared to many MT-inhibitory drugs, such as Vinblastine (VBL, 900 kDa), and their low molecular mass is expected to facilitate their delivery to target organs and tissues in animal models [34], iv) they inhibit cell growth in a broad array of cancer cell lines, including taxane-resistant derived lines [33–35]. The methods employed in those studies measured the global cell cycle profiles and the overall toxicity at the level of bulk cell population, but have limitations. Importantly, they neither identified the modes with which cell death occurred, nor did they clarify whether ATIs induce generalized cell death or whether cells become sensitized to death specifically during mitosis. Here we take a step further and investigate in depth the response of cancer cell lines to five selected ATIs that proved promising in previous screening assays. Combined high-resolution immunofluorescence (IF) and single cell time-lapse recording methods are used to pinpoint their specific features as mitotic inhibitors and cell death inducers, and contribute to identify effectors required for the cell death response.

## RESULTS

### ATI compounds with similar *in vitro* tubulin-binding activity arrest cell cycle progression with differential effectiveness in human cancer cell lines

**Figure 1:**
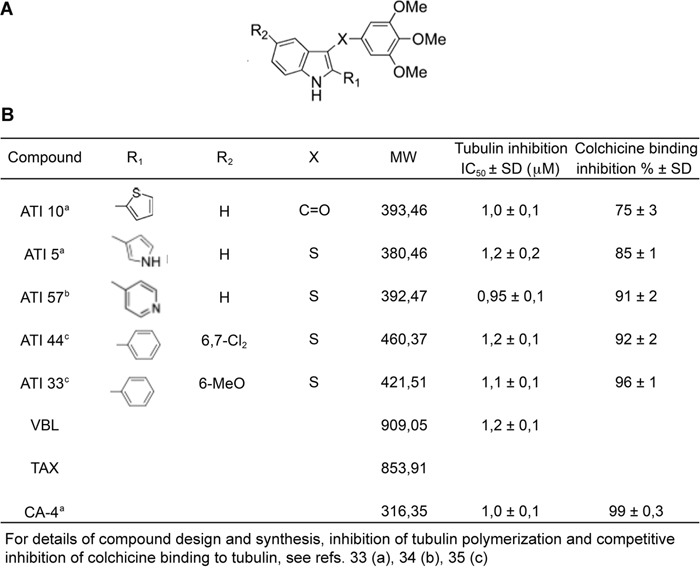
Properties of selected ATIs in human cells **A**. Schematic structure of the ATI scaffold. X, R1 and R2 represent positions in which specific substitutions were tested. **B**. Summary features of *in vitro* properties of five selected ATI molecules and of known MT-targeting drugs used in this work (for details of compound design and *in vitro* testing see 33-35).

The five ATIs examined in this work were designed based on the structure of the Colchicine-binding site on tubulin with molecular modeling back-up [37–38]. They were originally conceived as successive derivatives from a common structural scaffold (schematically shown in Figure 1A) and harbor substitutions at critical positions that confer improved metabolic stability and tubulin-binding activity [details of design and synthesis, as well as methods employed to measure tubulin polymerization inhibition and competitive inhibition of labeled Colchicine binding to tubulin, by each ATI, are given in 33-35]. The five molecules were previously examined in separate studies as members of large ATI subfamilies: they were found to inhibit tubulin polymerization with comparable IC50 at low micromolar concentrations *in vitro* and competitively inhibited Colchicine binding to tubulin (in all cases above 75% inhibition [33–35], data are summarized in Figure 1B for ease of comparison). All five ATIs inhibited cell growth in a variety of cancer cell lines, including multidrug resistant cells [33–35]. The underlying mechanism(s), however, were not examined in depth.

**Figure 2:**
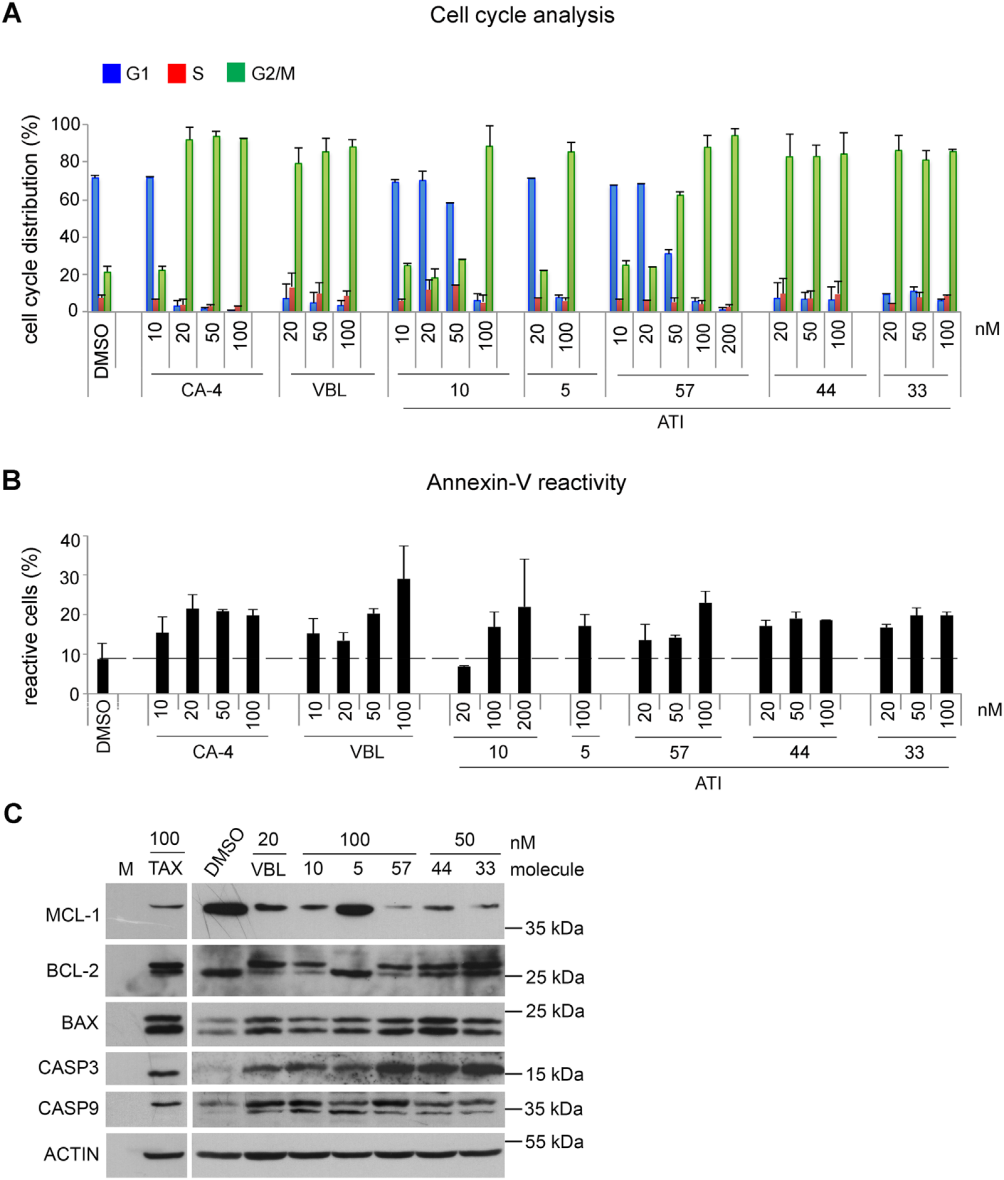
Dose-dependent induction of cell cycle arrest and cell death by ATIs in HeLa cultures **A**. Cell cycle profiles of HeLa cultures treated with known MT-targeting drugs and ATIs in the indicated concentrations, as determined by FACS assays of PI-stained cultures. Bars represent SD values calculated from 3 to 7 independent experiments per condition. **B**. FACS analysis of the overall induction of cell death by known MT-targeting drugs and ATIs, as determined by annexin V-FITC staining. As in A, bars represent SD values from 3 to 7 experiments per condition. **C**. Western blot of HeLa cell extracts after 24h of treatment with the indicated drugs: pro-survival and pro-apoptotic proteins in each treatment are shown.

To gain insight into their mode of action in restricting cancer cell growth, we first compared their effects on cell cycle progression in dose-response experiments. HeLa cell cultures were treated with increasing concentrations of each ATI for 24 hours (roughly covering the duration of one cell cycle), compared to VBL, a classical MT polymerization inhibitor with a long-record of therapeutic use, and Combretastatin A-4 (CA-4), also binding to the Colchicine site on tubulin and under test in clinical trials. By FACS analysis, all ATIs arrested progression of 4C cells (G2+M phases), yet clear differences in their effectiveness emerged (Figure 2A): ATIs 10 and 5 induced arrest in (G2+M) phases only when used 100 nM, while lower concentrations were ineffective. The pyridinyl derivative ATI 57 displayed some inhibitory effect already at 50 nM concentration, accumulating almost 60% of treated cells in the (G2+M) phases. ATIs 33 and 44 proved most effective cell cycle inhibitors, both inducing almost complete (G2+M) arrest already at 20 nM, comparable to VBL and CA-4 (Figure 2A).

In parallel FACS assays we evaluated the overall cell death by measuring the frequency of annexin V-positive cells in ATI-treated cell samples. ATIs displayed a parallel hierarchy in cell death induction to that seen for cell cycle arrest: both ATI 33 and 44 induced a significant fraction of annexin V-reactive cells already when used 20 nM and plateaued thereafter, again comparable to VBL and CA-4. ATI 57 induced a comparable rate of cell death when used in a five-fold higher concentration (100 nM). ATI 5 and ATI 10, when used 100 nM, also induced cell death, albeit consistently lower than other ATIs (Figure 2B). Together these results indicate that ATI molecules with similar tubulin polymerization inhibitory IC_50_ *in vitro* display in fact remarkably different efficacy in intact cells, revealed by their differential effectiveness in inducing (G2+M) phase arrest and cell death. Given that HeLa cells lack functional p53, the data in Figure 2B also imply that ATI-induced cell death is p53-independent.

We next investigated known cell death regulators in ATI-treated HeLa cell cultures. We first investigated pro-survival members of the BCL-2 family. MCL-1 and BCL-2 are both abundantly expressed in untreated healthy cells. In MT-damaged cells, down-regulation of MCL-1 protein abundance determines the length of mitotic arrest [39]. The abundance of MCL-1 is actually a critical determinant in the choice between either mitotic arrest, or slippage through the mitotic checkpoint and mitotic exit without proper chromosome segregation [40].

As expected, MCL-1 protein was abundant in DMSO-treated control cultures. VBL, TAX and ATIs dramatically down-regulated MCL-1 abundance, except ATI 5, which induced only a slight decrease in MCL-1 abundance. Thus, ATI-induced cell death requires MCL-1 down-regulation, as previously shown for classical MT-targeting agents [13, 39]. MCL-1 down-regulation (Figure 2C) was particularly strong in cultures treated with ATIs that induced effective (G2+M) phase arrest and cell death, i.e. 57, 44 and 33 (Figure 2B). We concomitantly observed changes in the electrophoretic mobility of BCL-2. TAX is known to induce BCL-2 phosphorylation, which triggers its inactivation and hence cell death [41, 30]. In our experiments BCL-2 migrates as a single band in control cultures (corresponding to the hypophosphorylated, anti-apoptotic form), whereas all treatments but ATI 5 induce the appearance of a doublet, in which the upper band (phosphorylated, inactive) is most abundant.

Parallel with BCL-2 phosphorylation, all drugs up-regulated the expression of the pro-apoptotic factor BAX. The increase was particularly evident with ATIs 57, 33 and 44, and it was comparable to that induced by TAX and VBL.

Mitotic cell death is associated with caspase-8 (CASP8), caspase-9 (CASP9) and caspase-3 (CASP3) activation [42], and pan-caspase inhibitors prevent cell death induction by MT-targeting drugs. CASP3, in particular, is activated by TAX [14], and also by ATI 5 in Jurkat lymphoblast cells [36]. We asked whether ATIs activate CASP9 or CASP3. Active CASP9 (37-35 kDa) is an apical caspase, activated via processing of pro-caspase 9 under death-inducing stimuli. We found that control cultures expressed only a faint trace of the 37 kDa active fragment, whereas TAX, VBL and all ATIs induced the active CASP9 form, indicating that CASP9 processing is a rapid response to ATI-dependent MT inhibition, including by the mildest ATI. We next analysed the effector caspase CASP3 using an antibody that selectively recognizes the 17 kDa processed fragment from procaspase-3. No processed product was detected in control cultures. In contrast, TAX, VBL and all ATIs consistently induced the processed, active CASP3 form. The increase in active CASP3 was particularly effective in response to ATIs 57, 33 and 44.

### Caspase-3 activity is required for ATI-induced mitotic cell death

The results indicate that all ATIs, but ATI 5, inactivate anti-apoptotic factors, particularly MCL-1, and activate pro-apoptotic factors, among which active CASP3 showed the most significant accumulation. *CASP3* gene mutations occur in various human cancers [43–44]. We therefore wished to assess whether CASP3 is not only implicated, but actually required for cell death induction by ATIs. To address this issue, we used MCF 7 breast carcinoma cell lines, which are functionally null for CASP3 expression, as they harbour a missense mutation in the *CASP3* gene that causes exon skipping and prevents *CASP*-3 mRNA translation [45]. These cells were compared to a paired isogenic MCF7 cell line, MCF7/CASP3, engineered to re-express CASP-3 [46]. We treated both cell lines with ATIs 5, 10 and 57 (100 nM). ATIs 33 and 44 were also tested at lower concentrations, based on their higher efficacy demonstrated in HeLa cells.

**Figure 3:**
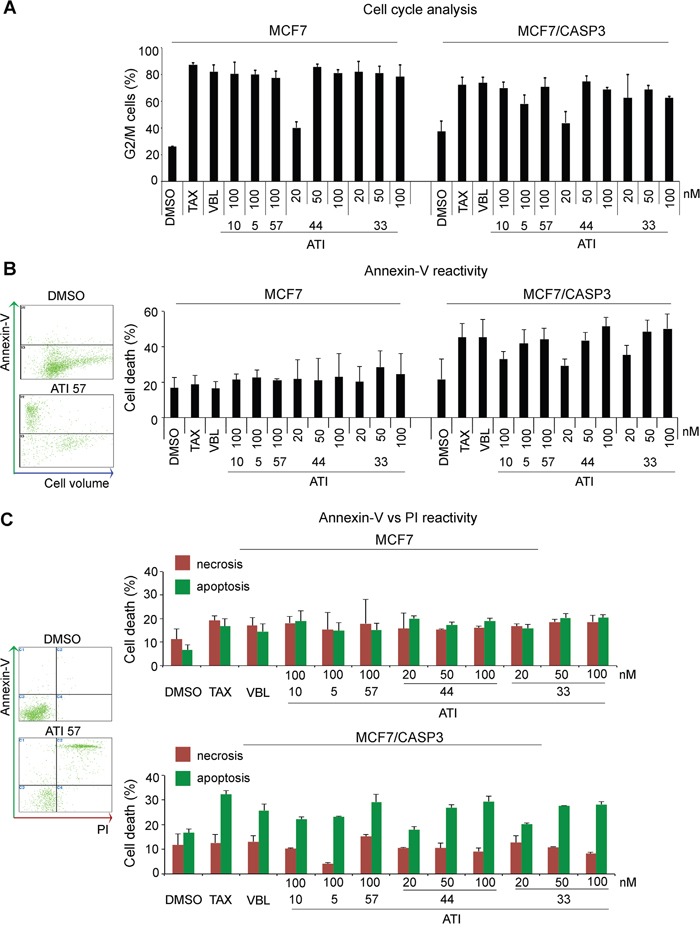
Caspase-3 requirement for non-necrotic cell death induction during mitotic arrest in MCF7 isogenic cell lines **A**. FACS analysis of cell cycle phases in PI-stained MCF7 and MCF7/CASP3 isogenic cell cultures: histograms represent the % of cells with 4C DNA content after 24 hours-exposure to the indicated drugs. Both isogenic cell lines undergo G2+M arrest regardless of CASP3 function. **B**. FACS analysis of overall cell death induction. Left, exemplifying biparametric panels of annexin V reactivity *vs* cell density (forward scattering axis) in control cultures (DMSO) and after exposure to ATI 57. The histograms show the frequency of annexin V-reactive cells in isogenic cell lines: only MCF7/CASP3-reconstituted cell cultures induce significant cell death in response to ATIs. **C**. Biparametric analysis of isogenic cell cultures double-stained for annexin-V and PI after ATI exposure. Exemplifying panels on the left show the distribution of viable cells with low values of both PI and annexin V (DMSO-treated control), and of cells undergoing necrosis (high PI, low annexin V values), or apoptosis-like death (high annexin V). MCF7/CASP3 reconstituted cell cultures induce significant apoptotic-like death in response to MT-targeting treatments. For all assays, means and SD values were calculated from three independent experiments for each cell line.

All ATIs arrested MCF7 cells in the (G2+M) phase (Figure 3A), similar to TAX and VBL. (G2+M) arrest was induced to a similar extent in the MCF7/CASP3 reconstituted cell line, confirming that the presence or absence of CASP3 does not influence the induction of mitotic arrest in the presence of damaged MTs [47]. A striking difference emerged instead in the distribution of dead cells between the isogenic cell lines. MCF7 cells are reported to activate an alternative CASP6 and -7 pathway after DNA-damaging agents [46, 48]. We found that all five ATIs induced a low level of cell death (revealed by annexin V analysis) in MCF7 cultures (Figure 3B). Increasing their concentration did not increase cell death any further (data not shown). Biparametric FACS profiles of annexin V and propidium iodide (PI) uptake in non-permeabilized cultures revealed that the low level of cell death observed in MCF7 cultures comprised cells with necrotic (PI permeable) and apoptotic-like features (high annexin V reactivity, low PI incorporation) in similar proportion. In MCF7/CASP3 cell cultures, instead, the death profile was shifted towards apoptosis, while the relative weight of necrosis was significantly reduced. The overall extent of cell death was generally higher in MCF7/CASP3 and varied with the specific ATI, with ATIs 44 and 33 being again the most effective compounds already at 50 nM. Thus, CASP-3, though being dispensable for mitotic arrest, is required for activation of non-necrotic cell death by ATIs.

### ATIs induce progressively severe dose-dependent MT damage in HeLa cells

The results thus far indicate that ATIs binding to the “Colchicine site”, and inhibiting tubulin polymerization with similar IC_50_ *in vitro*, are differentially effective in inducing mitotic arrest and cell death in cycling cells. To gain more insight into possible mechanisms underlying these differences, we compared the cytological effects of ATIs by immunofluorescence (IF) to alpha-tubulin, in HeLa cell cultures. All ATI treatments (24 hours) induced a high frequency of prometaphase-like figures with sparse condensed chromosomes (64-74%, depending on the ATI), while interphases were not visibly affected: thus, the (G2+M) enrichment depicted by FACS (Figure 2A) reflects genuine arrest in prometaphase, suggesting that all ATIs activated the spindle assembly checkpoint (SAC).

**Figure 4:**
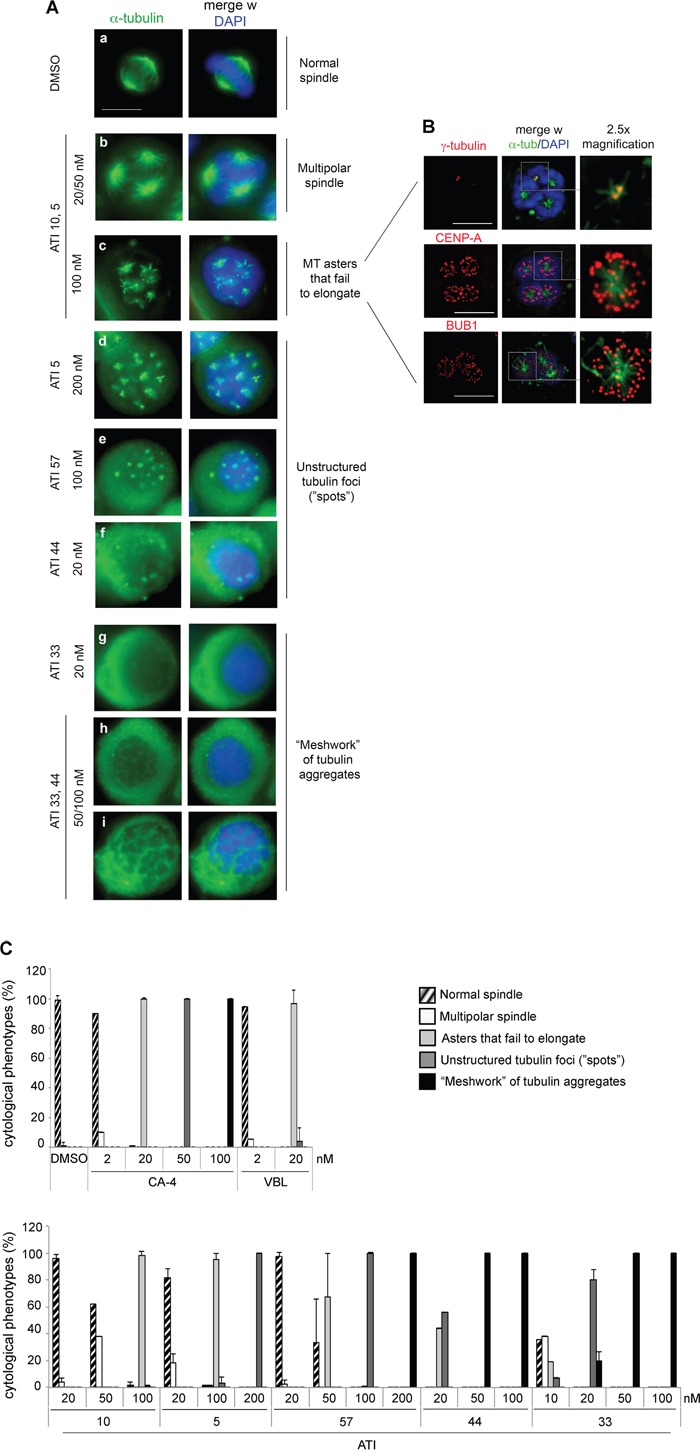
Mitotic phenotypes of increasing severity reveal a hierarchy in the strength of ATIs **A**. Examples of MT-inhibitory phenotypes induced by ATIs at the indicated concentrations in HeLa cell cultures. MTs are visualized by FITC-conjugated anti alpha-tubulin antibody (green) and chromosomes by DAPI staining of the DNA (blue). Mitotic phenotypes are arranged according to their increasing intensity. *Bar*, 10 μm. B. Deconvolved images of MT asters from HeLa cell cultures exposed to 100 nM ATI 10. Top panel, gamma-tubulin staining of MTOCs. Middle panel, CENP-A staining to visualize KTs. Bottom panel, Bub1 staining to depict KTs on which the SAC is active. In the central column, the merged images depict chromosomes stained in blue, alpha-tubulin in green and the third marker in red. For greater detail, the rightmost lane displays a 2.5x magnification of the boxed area. *Bar*, 10 μm. **C**. Frequency of increasingly severe MT-inhibitory phenotypes (histograms) induced by ATIs. Mean and SD values were calculated from 2 to 10 experiments per condition (at least 100 counted cells per experiment).

Individual ATIs induced cytological phenotypes of progressive strength (examples are shown in Figure 4A, and quantified in Figure 4C) by IF analysis of alpha-tubulin. Most severely damaged mitotic phenotypes were observed in cultures treated with ATI 33, in which most mitotic cells (negative for lamin B1 staining, not depicted) displayed condensed chromosomes and amorphous tubulin aggregates with a meshwork-like appearance (examples in Figure 4A, panels g-i). ATI 44 yielded a similar dramatic inhibition of tubulin assembly above 50 nM. Lowering its concentration to 20 nM induced a less severe phenotype, with sparse small tubulin foci visible as unstructured spots on a diffuse tubulin background (panel f). Similar patterns were observed with both CA-4 and VBL (20 nM, not shown), as well as ATI 57 (100 nM, panel e). Thus, ATIs 57, 44 and 33 induce extensive MT damage in a concentration-dependent manner.

ATI 10 (100 nM) induced the appearance of short MTs arranged in small radial asters (panel c). ATI 5 (200 nM) yielded sparse tubulin foci and tiny asters in variable proportions (panel d). Lower doses (20-50 nM) of both ATIs 5 or 10 caused milder phenotypes, most frequently multipolar spindles (panel b). Thus, the IF data collectively indicate that mitotic phenotypes generated by ATIs are dose-dependent and depict a hierarchical scale of MT damage induction in HeLa cells.

A priori, the IF results may suggest some target preference for ATI members: for example, ATI 10 may bind to exposed tubulin on early MT polymerization products and inhibit their elongation, whereas the six-atom ring ATIs (57, 33 and 44) may bind soluble tubulin and prevent its polymerization altogether. We examined phenotypes in cells exposed to varying concentrations of individual ATIs. Raising ATI 5 from 100 to 200 nM abolished the formation of asters and shifted the phenotype towards the formation of unstructured tubulin foci (“spots”) (Figure 4A, panels c-d). Conversely, lowering ATI 44 to 20 nM prevented the appearance of amorphous tubulin aggregates, i.e. the most severe phenotype, and also yielded tubulin foci (Figure 4A). Thus, the phenotypes in Figure 4A represent progressive stages of MT damage. Their concentration dependence suggests increasing scales of affinity of different ATIs, rather than distinct tubulin binding preferences.

We wondered whether the MT asters generated by ATIs 5 and 10 reflected the formation of supernumerary centrosomal MT-organizing centers (MTOCs) during prolonged treatment. Co-staining for alpha-tubulin (MTs) and gamma-tubulin (the most abundant MTOC component) revealed many asters in single prometaphase-arrested cells, while gamma-tubulin staining identified only two canonical MTOCs (Figure 4B). Thus, the formation of MT asters is not dependent on their physical association with centrosomal MTOCs, indicating that ATIs induce neither overduplication nor fragmentation of centrosomes. In a normal mitosis, kinetochores (KTs) stabilize MTs that come in contact with them and induce the nucleation of MTs that contribute to spindle formation. This pathway becomes particularly evident when centrosomal MT nucleation is impaired, and indeed several MT-damaging agents induce the formation of non-centrosomal asters [49–53]. We found that ATI 10-induced asters are composed of short MTs focused at one end, while the opposite end associates with CENP-A (the KT-associated nucleosomal histone) and with the SAC component BUB1 (Figure 4B). Thus, the short MTs interact with chromosomal KTs that stabilize them, but cannot grow to form a spindle. BUB1 signals on chromosomes indicates sustained SAC activation.

In wash-out experiments, cultures treated with the mild ATIs 5 and 10 showed faster progression from (G2+M) phase arrest compared to cultures treated with the six-atom ring ATIs 57, 33 and 44 (Supplementary Figure 1A). By IF analysis, mitosis resumption from MT asters yields grossly normal bipolar spindles, suggesting that the asters rapidly coalesce in two poles after ATI removal. Mitotic resumption from unstructured tubulin foci was slower and more prone to originate an abnormal mitotic apparatus, with persisting defects in chromosome segregation (Supplementary Figure 1B, compare mitosis resumption in the first three hours after wash-out of ATI 10, that had prevalently induced MT asters, to that of ATI 57-treated cultures, where mitotic cells had mostly arrested with unstructured tubulin foci. Higher resolution pictures are shown in Supplementary Figure 1C). Interestingly, cultures treated with ATIs 57, 44 and 33 showed largely defective reformation of the mitotic apparatus after wash-out, and still underwent cell death 24 hours after ATI removal (Supplementary Figure 1D).

### ATIs generate defective mitotic phenotypes in HT29 colorectal adenocarcinoma cells

The IF assays in HeLa cells suggest that the effectiveness of cell death induction by ATIs mirrors the severity of the mitotic inhibitory phenotypes that they induce. To assess the generality of this conclusion, we extended the experiments to HT29 colorectal adenocarcinoma cell lines, a widely used cellular model for its sensitivity to classical MT-inhibitory drugs [54].

**Figure 5:**
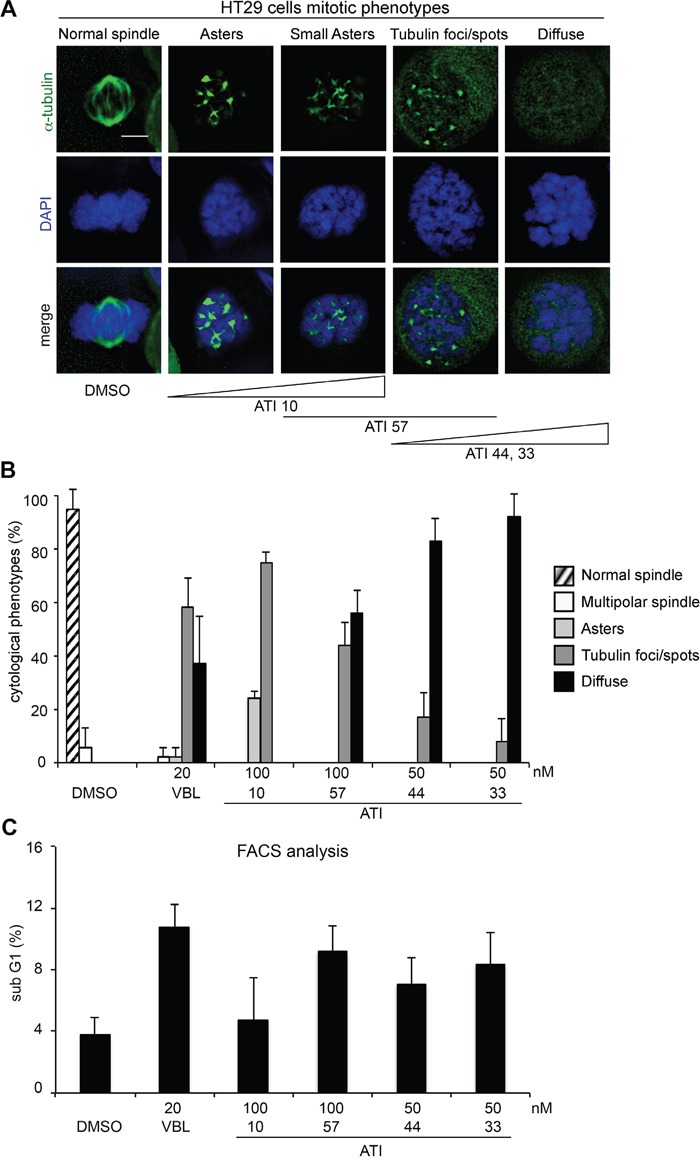
Abnormal MT phenotypes and cell death induction by ATIs in HT29 colorectal carcinoma cell cultures **A**. Representative examples of mitotic phenotypes induced by ATIs in HT29 cell cultures (MTs are visualized by FITC-conjugated anti-alpha-tubulin antibody in green and chromosomes by DAPI staining in blue). From left to right, mitotic phenotypes are arranged according to their increasing intensity. The prevalent induction of particular phenotypes by individual ATIs is indicated below. *Bar*, 5 μm. **B**. The histograms represent the frequency of the mitotic phenotypes illustrated in A after treatment with the indicated ATIs. **C**. The histograms represent the % of PI-stained cells with a <2C DNA content in ATI-treated HT29 cells after FACS analysis. For all assays, means and SD values were calculated from three independent experiments.

The results in Figure 5 confirm the same hierarchical scale of MT inhibitory strength as in HeLa cultures. Mitotic phenotypes detected by IF in HT29 cultures are exemplified in Figure 5A and quantified in Figure 5B. ATI 44 and, even more effectively, ATI 33 (both used 50 nM) prevalently induced prometaphase arrest with completely diffuse tubulin. ATI 57 induced mitotic defective phenotypes with either diffuse tubulin, or with tubulin foci, in comparable proportions. With ATI 10 tubulin foci became the prevalent phenotype, with some cells also displaying small MT asters. The hierarchical strength in tubulin inhibition demonstrated by ATIs in HT29 cells correlated with their ability to induce cell death (by FACS measurements of the sub G1 fraction, Figure 5C). ATI 10, which displayed the mildest mitotic inhibitory power, induced the lowest fraction of cell death compared to other ATIs. Instead, ATIs 57 (100 nM), 44 and 33 (the latter two used 50 nM) induced significant cell death, in the same range as VBL. Dose-response assays with ATI 10 (Supplementary Figure 2) showed that, in this sensitive cell line, the mild ATI 10 arrested cells in (G2+M) phases above 75 nM. MTs were generally damaged, preventing progression through mitosis and causing the (G2+M) accumulation detected by FACS. IF assays however revealed that treatment with 75, 100 or 150 nM ATI 10 yielded a remarkably different distribution of mitotic phenotypes: the formation of multiple MT asters was prevalent (73% of all mitosis-arrested cells) in samples treated with 75 nM, decreased to 26% in samples treated with 100 nM, and disappeared almost completely in samples treated with the 150 nM concentration, at which dose 90% of mitotic cells showed no discernible tubulin residual structure but sparse spots. That phenotype represents the most severe damage produced by ATI 10. Though still less severe than that produced by the six-atom substituted ATIs, it is clearly more predictive of cell death than the simple induction of (G2+M) arrest (Supplementary Figure 2B). These experiments confirm and extend the results obtained in HeLa cells. We conclude that ATIs can effectively block mitotic progression, yielding (G2+M) arrest, by inducing mitotic MT inhibition to a varying extent. That however does not necessarily activate death signals, as cell death induction is associated with inhibitory phenotypes of increasing cytological severity.

### ATI members induce distinctive cell fate profiles in time-lapse recording assays

**Figure 6:**
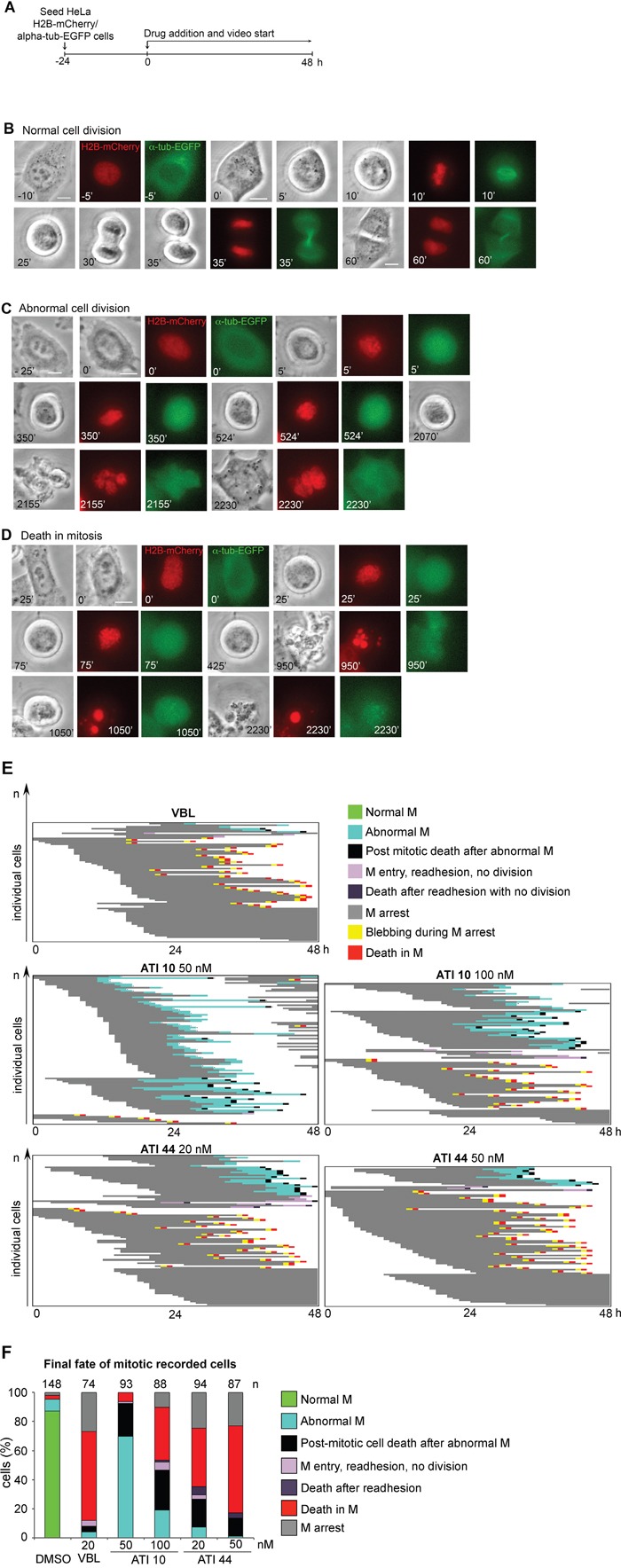
Single cell analysis of video-recorded cells in HeLa/H2B-mCherry/alpha-tubulin-EGFP treated cultures **A**. Time-lapse protocol used to record HeLa/H2B-mCherry/alpha-tubulin-EGFP cultures treated with MT-targeting drugs. Cultures were recorded form the time of drug addition (time 0) over the next 48 hours. **B**. Representative control cell (DMSO-treated) undergoing normal division. Phase contrast pictures, chromosomes (H2B-mCherry) in the red fluorescence channel, and alpha-tubulin in the green fluorescence channel are shown. **C**. Cell exposed to 50 nM ATI 10, showing prometaphase delay followed by aberrant mitotic exit without division, readhesion and generation of a multinucleated cell. **D**. Still frames depicting cell death during mitotic arrest in a cell treated with 100 nM ATI 10. *Bars*, 10 μm. **E**. The panels represent the fate of single cells in cultures treated as indicated. Each line depicts a single HeLa/H2B-mCherry/alpha-tubulin-EGFP cell. **F**. The histograms represent the frequency of terminal fates recorded in control (DMSO) and treated cultures. The number of recorded cells in two independent time-lapse assays are shown above each histogram.

To assess more directly the link between mitotic MT damage and cell death induction, we decided to record live cell cultures during exposure to ATIs by time-lapse. In initial experiments we used a HeLa cell line expressing H2B-red Cherry histone to visualize chromosomes and EGFP-tubulin to visualize MTs [55]. Cells were exposed to ATIs that induce either mild (ATI 10) or strongly defective cytological phenotypes (ATI 44) by IF, or VBL for comparison, and were recorded from the time of drug administration (protocol in Figure 6A). That enabled us to focus on early time points. Under all conditions, cells progressed from whichever cell cycle stage they were at the time of drug administration up to mitotic entry. Most control cells accomplished mitosis in less than 2 hours (see for example Figure 6B): because cells regularly enter and exit mitosis in DMSO-treated control cultures, the mitotic index remains constant from the time of DMSO administration (time 0) over the next 32 hours (Supplementary Figure 3A). Cultures exposed to ATI 44 (50 nM), ATI 10 (100 nM), or VBL entered mitosis with a timing that was practically indistinguishable compared to controls, but remained blocked soon after round up in early prometaphase, and progressively accumulated in that stage (Supplementary Figure 3A). VBL and ATI 44 induced sustained mitotic arrest (Supplementary Figure 3B): treated cells that entered mitosis early during the video, i.e. 0 to 4 hours after drug administration, remained arrested throughout the recording time (over 20 hours). A fraction of cells were arrested 10 to 20 hours, and a minor fraction, corresponding to cells that entered mitosis late during the video, were arrested for 5 hours or less. ATI 10 induces a slightly lower fraction of stably arrested cells (>20 hour-long arrest). Given that the drugs do not induce significant differences in mitotic entry, the data indicate that ATI 10 tends to induce less sustained mitotic arrest, with some cells eventually readhering to the culture dish after either aberrant or failed division after prolonged mitotic delay (see for example Figure 6C).

To gather information on the terminal fates of the cells we recorded single cell fates induced by ATIs 10 and 44, at two different concentrations, during 48 hours from the time of drug addition. The recorded individual cell fates are plotted in Figure 6E. The EGFP fluorescence associated with depolymerizing tubulin *in vivo* was not amenable to detailed resolution of the MT damage type in video-recording, yet interesting information was obtained. 50 nM ATI 10 induced most treated cells to divide aberrantly. Some daughter cells activated cell death after the abnormal division, yet a significant fraction re-entered the cell cycle and divided abnormally again. Elevating the concentration of ATI 10 to 100 nM remarkably reduced that cell population: abnormal divisions still occurred, yet many cells proficiently activated mitotic cell death (Figure 6D shows a cell undergoing death during sustained mitotic arrest, with intensely fluorescent fragmented nuclei). In cultures treated with ATI 44 (20 nM) abnormal divisions still took place, but were almost invariably followed by death of the daughter cells. A fraction of treated cells failed to divide and, after prolonged mitotic arrest, readhered and thereafter underwent death. Raising ATI 44 to 50 nM suppressed many intermediate cellular fates and induced death in most mitotic-arrested cells. These initial recording assays link the capacity of ATIs to induce cell death to the severity of induced MT damage (Figure 4) and hence the induction of sustained mitotic arrest (Figure 6E). We therefore recorded systematically the fate of cells treated with each of the five ATIs under the same conditions under which we had characterized their MT-damaging strength (Figure 4). Because the use of EGFP-tubulin might have added an additional level of phototoxicity, complicating the interpretation of the effects of the ATIs over MTs, we used a HeLa-derived cell line expressing only H2B-GFP to visualize chromosomes.

**Figure 7:**
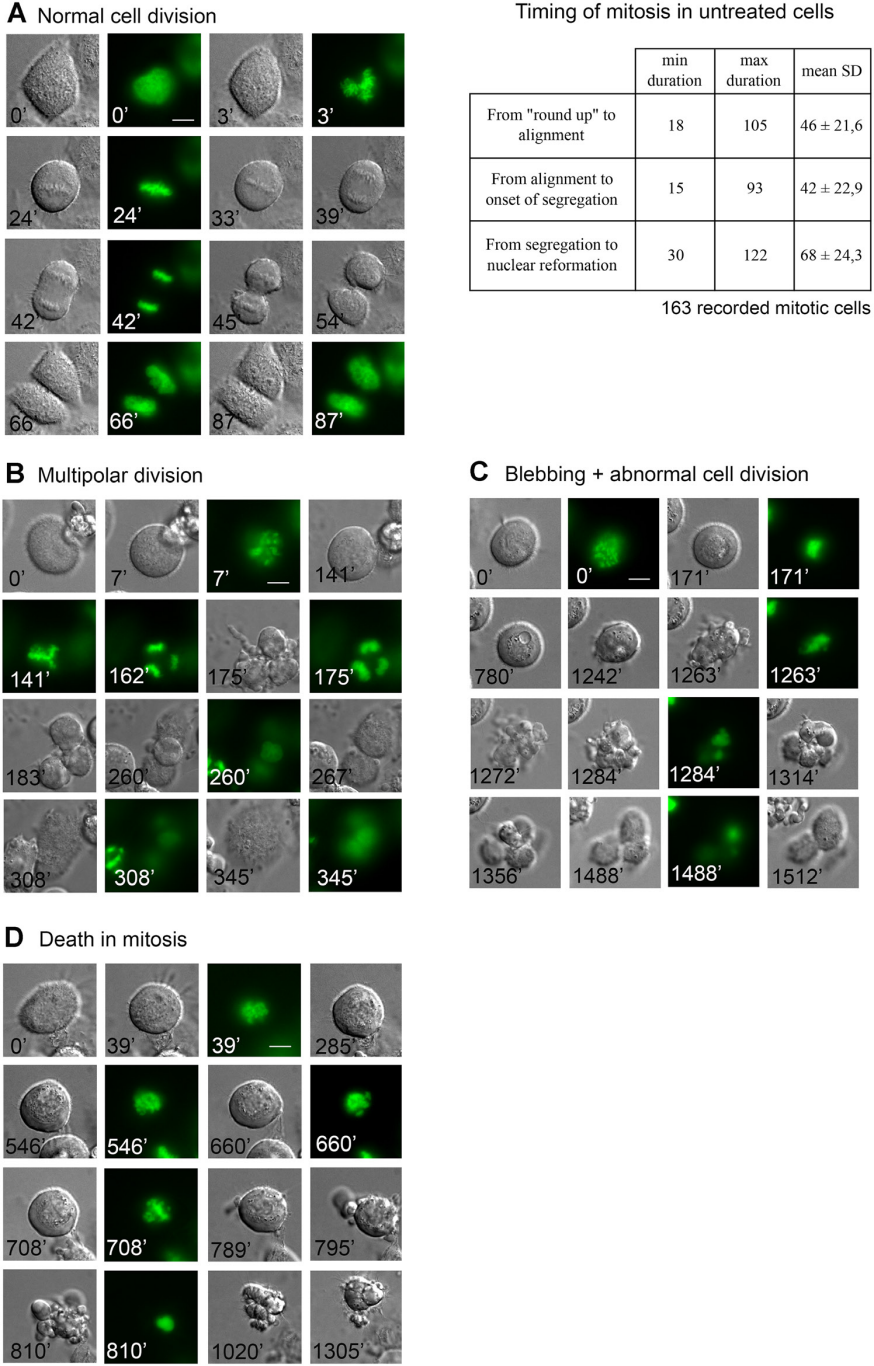
*In vivo* imaging of mitotic cell fates induced by ATIs in HeLa/H2B-GFP treated cultures **A**. Still frames from a recorded HeLa/H2B-GFP cell undergoing mitosis with a normal timing. Cell division is complete in about one and a half h after the onset of mitotic round-up (taken as time 0). The timing of mitotic phases recorded from 163 cells is shown on the right. **B**. Still frames from an exemplifying videorecorded cell exposed to ATI 10 showing transient delay in prometaphase, then reaching multipolar alignment and undergoing aberrant mitotic exit followed by fusion to form a single multinucleated cell. **C**. Still frames from a videorecorded HeLa/H2B-GFP cell treated with ATI 10 undergoing prolonged delay in prometaphase (up to 1500 min from the onset of recording), followed by intensely dynamic blebbing and abnormal mitotic division; the two unequal mitotic products eventually readhere and appear to reenter interphase. **D**. Still frames from a videorecorded HeLa/H2B-GFP cell treated with ATI 33, exemplifying death during mitotic arrest.

Figure 7A shows an untreated H2B-GFP/HeLa cell dividing normally. Occasionally, some cells that reached mitosis late during videorecording divided along a multipolar axis (see for example Figure 7B), or left unresolved chromatin bridges between daughter cells. A small fraction of cells remained arrested in mitosis throughout the video, possibly reflecting hindrances in completing mitosis during medium exhaustion. We next recorded treated cultures, and included TAX for comparison, the effects of which are extensively characterized [28, 56–57].

**Figure 8:**
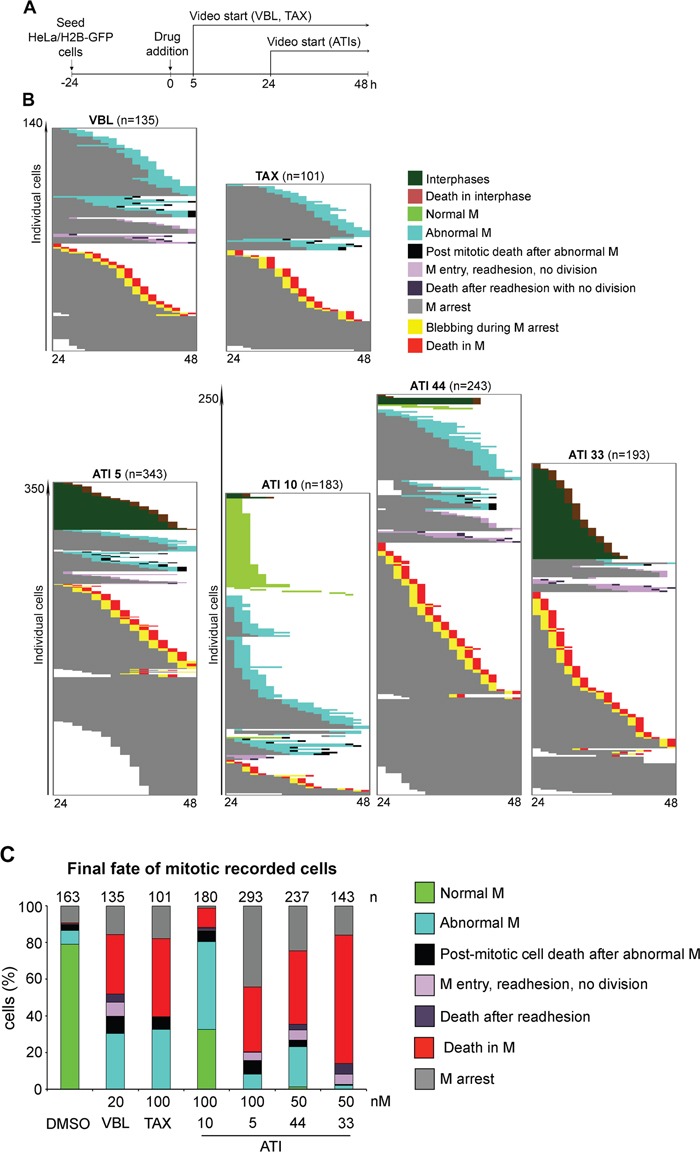
Single-cell analysis of video-recorded cell fates induced by ATIs **A**. Protocol for cell fate recording in HeLa/H2B-GFP cultures treated with MT-targeting drugs. **B**. The panels represent the fate of individual cells that either enter mitosis, or die in interphase, in samples treated with the indicated drugs. Each line in the panels depicts a single HeLa/H2B-GFP cell in the 24-48 hours time window post-treatment. Cells were recorded in at least 3 time-lapse assays for each ATI and 2 for VBL and TAX; total number of cells are indicated. The ATI 5 panel is depicted in a 50% reduction. **C**. The histograms represent the frequency of terminal fates in cells that enter mitosis (numbers are shown above each histogram) in control (DMSO) and treated HeLa/H2B-GFP cultures.

With either TAX or VBL treatment (protocol in Figure 8A), most cells that reached mitosis during the first 24 hours of recording became stably arrested soon after round up, then underwent diversified fates (Figure 8B, left panel, VBL; right panel, TAX). A fraction (16-18% with both TAX and VBL) remained durably arrested throughout the video. Many cells activated the death pathway during mitotic arrest. A fraction (over 30% with either drug) exited mitosis after a variable delay and generated abnormal products with either unsegregated, or randomly segregating chromosomes. Many of these abnormal mitotic products died in the next interphase, but about 30% remained apparently viable. These experiments define the window from 24 to 48 hours after drug addition as most informative. They also identify distinctive phenotypes that followed MT inhibition, but not MT hyperstabilization. Indeed, VBL triggered an array of cell fates in cells that entered mitosis, yet with specificities compared to TAX: first, VBL-arrested prometaphases underwent rapid changes in cell shapes and projection of cytoplasmic protrusions that were not observed in TAX-treated cultures. Second, some VBL-treated cells that experienced a long prometaphase delay failed to initiate chromosome segregation and eventually readhered to the culture dish and decondensed chromosomes in an undivided cytoplasm (as previously described in the H2B-mCherry/EGFP-tubulin cell line in Figure 6). Both processes, i.e. the cytoplasmic protrusions and the readhesion without division, are specific responses to MT depolymerization and are not seen with TAX.

We finally recorded ATI-treated samples, focussing on the 24-48 hours after treatment to minimize phototoxicity (Figure 8A). The lower panels in Figure 8B represent the fate of single recorded cells. These assays confirmed that ATI 10 transiently arrested mitosis, yet only a relatively small fraction of cells (about 11%) underwent death in mitosis, preceded by a highly intense blebbing activity (see Figure 7D). About one third of the cells resumed mitosis after prolonged prometaphase delay. About 14% of these cells divided abnormally after prometaphase arrest and generated aberrant products (see for example Figure 7C), a fraction of which died in the next interphase.

ATI 5 (100 nM) yielded more diversified cell fates. Almost half of the cells that entered mitosis remained durably arrested in prometaphase, and about 35% died during prometaphase arrest. A fraction eventually bypassed the arrest and divided aberrantly, often followed by fusion of the cell division products (Figure 7C). We also noted that some mitotic cells (about 5%) readhered after prolonged prometaphase, as seen with VBL. These cells are tetraploid, hence genetically similar to the group that re-fused after segregation. Time-lapse recording reveals however some functional diversity between the re-adhesion phenotype (no attempt of chromosome segregation with re-adhesion in prometaphase) and the class represented in Figure 7C (aberrant chromosome segregation in anaphase, followed by the failure to terminate cell division and fusion of the generated products): different stages of mitosis are impaired, respectively early (prometaphase) and late (final cytokinesis), possibly reflecting differential mitotic MT inhibition in individual cells.

Both ATI 33 and 44 (50 nM, yielding the formation of tubulin aggregates in IF assays) arrested many cells in early mitosis in the recording assays. Prometaphase-arrested cells underwent rapid formation and projection of protrusions, more pronounced and prolonged than recorded with other ATIs. This often evolved in mitotic death: over 40% of cells died in mitosis with ATI 44 and as many as 70% with 33, which consistently proved the most severely MT-damaging ATIs. Some cells treated with both drugs either re-adhered while in prometaphase, or divided aberrantly, often followed by death in the next interphase. A fraction of the cells (24% with ATI 44 and 16% with 33) were still stably blocked in mitosis after 48 hours of treatment. ATI 33 yields a lower frequency of abnormal divisions and higher death in mitosis compared to ATI 44. The frequency of the terminal cell fates in treated populations is schematized in Figure 8C.

In summary, all ATIs arrest mitosis and establish conditions for an array of possible fates. The recording data depict specific differences in the ratio of mitotic death to prolonged mitotic arrest, with the latter potentially exiting in abnormal mitotic division, re-adhesion to form multinucleated cells, or post-mitotic death. They depict a parallel between the evolution of cell fates and the type of MT cytological phenotype induced by distinct ATIs and identify death in mitosis as the prevalent fate induced by the 6 atom-ring ATIs. Collectively, the data indicate that the severity of MT damage (Figure 4) is a better predictor of mitotic cell death induction by tubulin inhibitory drugs compared to their cell cycle effects, which in can be apparently similar (Figure 2).

## DISCUSSION

Tubulin-targeting molecules are actively being designed and assayed in various types of cancer cell lines, yielding consistent arrest of cell cycle progression, confirming that mitosis remains a valuable cancer therapeutic target. It is proposed that the “ideal” anti-mitotic drug should arrest cancer cell division and induce death in mitosis to prevent the generation of genetically unbalanced, hence unstable, cells - which may cause aggressive tumor progression and relapse over time. The issue of the sensitivity of cancer cells to anti-mitotic drugs is being intensely investigated. Both the genetic background of the cancer cells, and the expression profile of genes encoding MT-regulatory factors, influence the ultimate outcome of MT-targeting molecules [12]. The development of single cell methods also growingly underscores ample variations in the response of single cells, even with identical genetic background [26–27, 58], adding an additional layer of complexity in predicting the cancer cell response.

**Figure 9:**
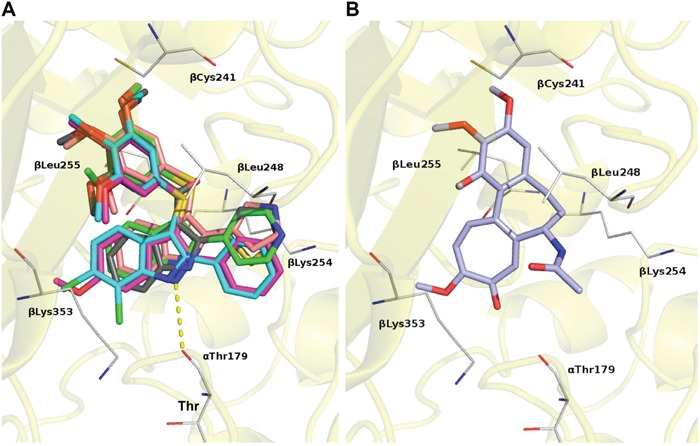
Predicted binding modes for ATIs and Colchicine by molecular modeling **A**. Modeling of the ATI-binding site on tubulin. Tubulin is depicted as a yellow ribbon and specific ATI compounds as sticks of different color: ATI 5, pink; ATI 10, gray; ATI 33, magenta; ATI 44, cyano; ATI 55 green. H-bond is depicted as a dotted yellow line. **B**. Modeling of the Colchicine-binding site on tubulin. Colchicine is depicted as a lilac stick. Residues involved in contacts are shown as white lines.

Here we have selected five structurally related ATI molecules endowed with similar *in vitro* tubulin binding properties, similar competitive inhibition of Colchicine biding to tubulin, and grossly similar growth suppressive effects in conventional cell toxicity assays [33–35]. Such compounds share a consistent binding mode at the Colchicine site. Docking experiments (Figure 9) highlight some common interactions: an H-bond with αThr179; a polar contact between βCys241 and methoxy moieties; the trimethoxyphenyl group formed hydrophobic interactions with βLeu248, and βLeu255; the 5- and 6- member rings at position 2 of the indole set up hydrophobic interactions with the βLys254 and βLeu248 side chains; the indole ring established contact with βLys353 side chain (Figure 9A). The binding of Colchicine is modeled in Figure 9B for comparison.

All ATIs yielded an accumulation of cells with a 4C genomic content, as a result of mitotic arrest or delay in treated cell populations. Different ATIs displayed however a differential MT inhibition capacity in mitotic cells, that was hierarchically distinguishable at the cytological level in dose-dependent assays. It is possible, in principle, that different ATIs have differential pharmacokinetics properties at the single cell level, and that this contributes to the observed differences both between structurally similar ATIs, and in individual cells exposed to the same ATI. At the cytological level, we found that ATIs carrying a five-atom ring (ATI 5 and 10) allowed some degree of MT polymerization, with the formation of either multipolar spindles (lacking structural cohesion at the spindle pole level), or asters of short radially arranged MTs. The asters were associated with KT proteins through the MT outer ends, suggesting that they reflect the activation of the MT nucleation pathway driven by KTs. Raising the concentration of the aster-inducing ATIs inhibited tubulin polymerization more extensively, with only sparse foci remaining visible. ATI molecules carrying a six-atom ring at position 2 of the indole, particularly ATIs 33 and 44, proved highly effective tubulin inhibitors, preventing MT assembly in the 20-50 nanomolar range, similar to VBL. Higher concentrations yielded a meshwork of aggregate-like structures. Thus, although *in vitro* properties would not have predicted dramatically different MT inhibitory capacity for these molecules, IF analysis depicted significant differences in cells. These differences were consistently observed in HeLa cervix carcinoma and HT29 colorectal adenocarcinoma cell lines. Retrospectively, the alpha-tubulin IF pattern induced by specific ATIs provides a more accurate basis for predicting the treatment outcome in dividing cells. We suggest that the appearance of tubulin aggregates, or unstructured tubulin spots, or MT asters, which directly visualize the extent of the cell growth-inhibitory properties of ATIs, can provide a rapid test to predict the effectiveness of MT-inhibitory drugs at any given concentration.

Time-lapse recording assays depicted variations within HeLa cultures in response to one same compound. ATIs generally activated a proficient SAC response, and most treated cells induced prolonged arrest in prometaphase, with condensed chromosomes and no, or defective, MTs. ATIs that induced a relatively “mild” cytological phenotype induced only a transient mitotic delay. Eventually cells exited mitotic arrest along two major processes: i) resumption of mitosis in the absence of a functional mitotic apparatus, followed by aberrant segregation and generation of aneuploid cells, or ii) failure to complete mitotic division and cytokinesis, followed by readhesion with an undivided cytoplasm and generation of a polyploid cell. After slippage, a fraction of the newly generated cells die in the following interphase. It is now known that MT-targeting drugs activate death pathways that rely on the activation of a DNA damage response during prolonged mitotic delay [59–62], which triggers factors important for post-mitotic death. It is unclear how post-mitotic death is induced in HeLa cells, which lack functional p53 due to expression of the E6 inactivating antigen. Our time-lapse assays recorded a frequent failure of segregation of H2B-GFP labeled-chromosomes, suggesting that heavy mis-segregation and genetic unbalance of the cell division products causes their loss of viability, regardless of p53 function.

The six atom-ring ATIs induce rare mitotic slippage and, consequently, rare post-mitotic cell death events. They rather activate death in prometaphase-arrested cells, especially ATI 33. The effectiveness of this pathway in HeLa cells suggests that cell death is p53-independent. Western blot assays indicate that ATIs induce the activation of BAX and CASP3 and -9, as well as massive downregulation of MCL-1 and phosphorylation (an inactivating modification) of BCL-2. MCL-1 downregulation and CASP3 activation are particularly significant in cultures treated with ATIs 57, 44 and 33, suggesting that ATI-dependent cell death relies particularly on mechanisms mediated by these factors. Indeed, we find that CASP3 is essential for non-necrotic death induction in ATI-treated cells. Interestingly, the bis-indole KAR-2, a derivative of VBL, causes a rise in intracellular Ca(2)+ levels [63], similar to other MT-destabilizing agents. Ca(2)+ release can directly modulate cell death. In addition, Ca(2)+ levels can fragilize MTs and hence cooperate with MT-inhibitory molecules in disrupting mitotic spindle function and structure, thus reinforcing the cell death signals initiated by MT damage. It might be speculated that ATIs can also differentially modulate intracellular Ca(2)+ release, which might in turn modulate cell death. On the whole, the results presented here indicate that the severity of induced MT damage by individual ATIs is a more effective trigger of mitotic cell death than the length of mitotic arrest per se, suggesting that the threshold for emanating death signals by MT damage can be predicted from accurate IF characterization of the cytological phenotypes.

Time-lapse recording is extensively used to depict the unfolding of dynamic programs and/or response to particular conditions, but is employed surprisingly poorly in drug design and validation studies, although the trend is increasing. Studies of new drugs typically examine whole cell-level responses in cultured cells, e.g. the overall cell cycle and cell death profiles, and/or biochemical studies of the chosen target, then often move to preclinical studies in animal models. That typical protocol misses out information on the dynamics and heterogeneity of the response of individual cells. For example, assays of the mitotic kinase Aurora-A inhibitor MLN8237 (Alisertib), did not include videorecording data prior to undergoing pre-clinical and phase I-III clinical trials. Recording methods later depicted heterogeneous cell fates, with possibly detrimental consequences (low mitotic death, high aneuploidy induction), that had not been originally appreciated [64]. The present results further highlight methodological caveats in bulk cell population analyses. Coupling mitotic phenotypes and single-cell recording data provides reliable informative tools to characterize potentially interesting compounds that may undergo further levels of validation.

In conclusion, the data presented in this work pinpoint the effectiveness of members of the six-atom ring ATIs in triggering death in mitosis in the nanomolar range, comparable to, or higher than, canonical MT-targeting inhibitors such as VBL, yielding little mitotic slippage or multipolar divisions. These ATIs can represent a valuable addition to increase the molecular diversity of MT-targeting agents.

## MATERIALS AND METHODS

### Compounds

ATIs were synthesized at the Drug Design and Synthesis Center of Sapienza University of Rome as described previously [33–35]. VBL (sulfate salt, Sigma V1377), CA-4 (Sigma C7744) and TAX (Selleck S1150) and all ATIs were dissolved in DMSO (Sigma, 472301; 0.1% final concentration). In comparative studies, ATIs were used 100 at nM unless otherwise indicated. TAX was used at 100 nM. VBL and CA-4 at 20 nM. In all experiments control cultures were treated with 0.1% DMSO.

### Cell cultures and treatment

HeLa cervix carcinoma, HeLa/H2B-mCherry/alpha-tubulin-EGFP [55], HT29 colorectal adenocarcinoma and MCF7 breast carcinoma cell lines were from ATCC (American Tissue Culture Collection). The MCF7/CASP3 cell line reconstituted with caspase-3 was a kind gift from Dr. Christopher J. Froelich (North Shore University, Health Systems Research Institute, Evanston, IL, USA) [46]. The HeLa-derived cell line stably transfected with H2B-GFP was described [65]. Cell lines were all grown in Dulbecco's Modified Eagle medium (D-MEM) supplemented with 10% fetal calf serum (20% for MCF7/CASP3), 2% L-glutamine and 2% penicillin/streptomycin, at 37°C with 5% CO_2_. In order to maintain HeLa/H2B-mCherry/alpha-tubulin-GFP cell line, 0.5 μg/ml puromycin (Clontech, 631305) and 0.5 μg/ml G418 (Sigma, A1720) were used. In experiments with all cell lines 300,000 cells were plated in 9 cm^2^-culture dishes and treated with MT-targeting compounds for 24 hours unless indicated otherwise.

### FACS analysis

Cell cycle phase distribution was analyzed after incubation with propidium iodide (PI, Sigma P4170). All parameters (FS, SS and FL-3) were acquired in a linear amplification scale. Cell aggregates were gated out on the bi-parametric graph FL-3lin/Ratio. Cell death was analyzed after incubation with Annexin V-FITC (Immunofluorescence Science, IK-11120), either alone or in combination with PI. In the latter case, both FL-1 (FITC) and FL-3 (PI) were acquired in a log amplification scale. Cell samples were analyzed in a Coulter Epics XL cytofluorometer (Beckman Coulter) equipped with EXPO 32 ADC software. At least 10,000 cells per sample were acquired. Data were processed using EXPO 32 ADC or Win MDI software.

### Western blotting

HeLa cell cultures were exposed to either solvent (0.1 % DMSO), or TAX, VBL and each of the ATIs at the indicated concentrations. After 24 hours protein extracts were separated through SDS-polyacrylamide gel electrophoresis, transferred to nitrocellulose filters, blocked and incubated with the following primary antibodies, all screened using TAX-treated extracts: mouse anti-MCL-1 (22) (sc-12756, 1:200 over night), mouse anti-BCL-2 (C-2) (sc-7382, 1:500 over night), mouse anti-BAX (B-9) (sc-7480, 1:500 over night) and goat anti-actin (I-19) (sc-1616, 1:400), all from Santa Cruz Biotechnology; rabbit anti-cleaved Caspase-3 (Asp175) (#9661, 1:500 over night) and rabbit anti-Caspase-9 (#9502, 1:1000 over night) both from Cell Signaling Technology (Danvers, MA, USA). Horseradish peroxidase-conjugated secondary antibodies (goat anti-mouse 1:10000 for anti-MCL-1 and anti-BAX or 1:2000 for anti-BCL-2, goat anti-rabbit 1:2000 and donkey anti-goat 1:5000 from Santa Cruz Biotechology) were revealed with enhanced chemiluminescence (ECL) or ECL plus (GE Healthcare, Chalfort Saint Giles, UK).

### Immunofluorescence

After treatment, cells grown on sterile poly-L-lysine (Sigma P4832) coated coverslips were fixed in 100% methanol (6 min, -20°C). Primary antibodies were: alpha-tubulin, either unconjugated (B5-1-2) or FITC-conjugated (DM1A), both from Sigma; Bub1 (Millipore, MAB3610); CENP-A (Abcam, mouse monoclonal 13939); gamma-tubulin (Sigma-Aldrich, GTU-88). Secondary antibodies were conjugated to FITC or Cy3 (Jackson Immunoresearch Laboratories), Texas Red (Vector Laboratories), or AMCA (Santa Cruz Biotechnology). DNA was stained with 0.05 μg/ml DAPI (Sigma D9542) and coverslips were mounted in Vectashield (Vector Laboratories).

### Microscopy

IF processed cell preparations were examined under an epifluorescence microscope Nikon Eclipse 90i equipped with a QICAM Fast 1394 camera (QImaging) using the NIS-Elements AR 3.2 software (Nikon). Single cell images were routinely taken using immersion oil 100x objectives with NA 1.3. 0.3 or 0.6-μm z-serial optical sections were processed using either the “Deconvolution module” (NIS-Elements AR3.2, Nikon) or the “AutoQuant module” (NIS-Elements AR4, Nikon). Processed images using deconvolution algorithms were flattened using the Extended Depth of Focus tool.

### Time-lapse recording

HeLa/H2B-GFP and HeLa/H2B-mCherry/alpha-tubulin-EGFP cells were seeded in glass-bottomed 3.5 cm plates (Ibidi 81158) or μ-Slide (chambered coverslip) with 4/8 wells (80426/80821, IbiTreat, Ibidi). During recording, cell cultures were kept at 37°C in a temperature- and CO_2_ controlled microscope stage incubator (Basic WJ, Okolab). Cultures were recorded under an automated inverted microscope (Ti Eclipse, Nikon) equipped with a DS-Qi1MC camera, an Intensilight C-HGFIE lamp, and the NIS-Elements 3.2 software (all from Nikon). Phase-contrast (60x 0.7 NA) or immersion oil (60x 1.4 NA) objectives were used. Most recording experiments were carried out for 48 hours as indicated in the text. In the HeLa/H2B-GFP cell line, differential interference contrast (DIC) images were taken every 3 min and GFP images every 20 or 21 min. In the HeLa/H2B-mCherry/alpha-tubulin-EGFP cell line, Phase (Ph) images were taken every 3 or 5 min, GFP and mCherry images every 25 min.

### Molecular modeling

Docking experiments were carried out following a previously reported procedure [33–35, 37]. Figure 9 was created using Pymol (www.pymol.org).

## SUPPLEMENTARY MATERIALS FIGURES



## Abbreviations

ATI: arylthioindole
CA-4: combretastatin A-4
FACS: fluorescence-activated cell sorter
IF: immunofluorescence
KT: kinetochore
MT: microtubule
MI: mitotic index
MTOC: microtubule organizing center
TAX: Taxol
VBL: Vinblastine.
