# Supplementary material for: Mitotic cell death induction by targeting the mitotic spindle with tubulin-inhibitory indole derivative molecules
Source: Oncotarget. 2017 Feb 1;8(12):19738–59. doi: 10.18632/oncotarget.14980 (PMC5386718; doi:10.18632/oncotarget.14980)
Supplement: Supplementary file 1 [file oncotarget-08-19738-s001.pdf]

# Mitotic cell death induction by targeting the mitotic spindle with tubulin-inhibitory indole derivative molecules

## SUPPLEMENTARY FIGURES

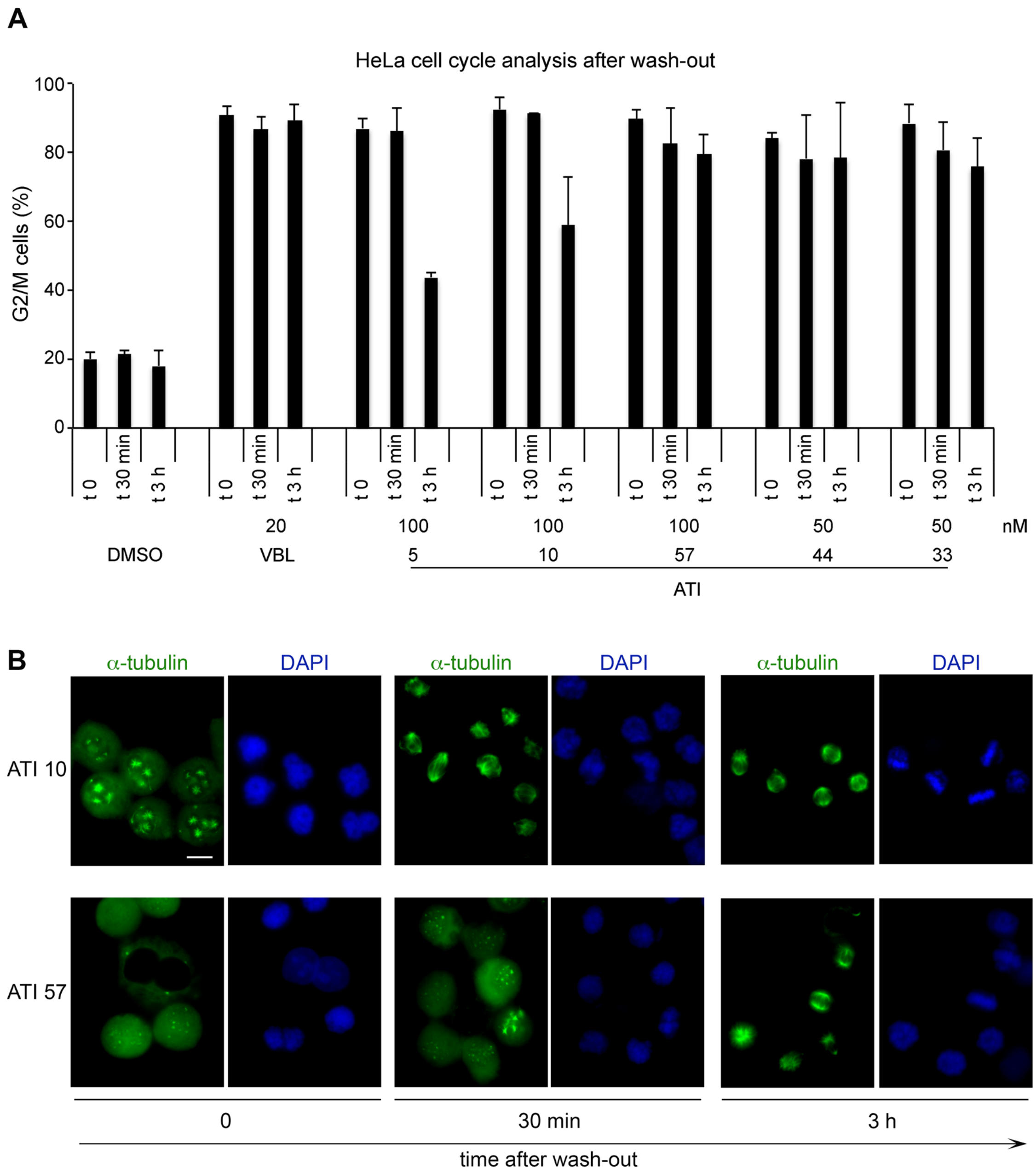


**Supplementary Figure 1: FACS and IF analysis to visualize the recovery from cell cycle-arrest and MT-inhibition after ATIs removal. A.** FACS analysis of PI-stained HeLa cells: histograms display % of G2/M cells after 24 hours of treatment (t0) and after drug removal (30 minutes and 3 hours), for the indicated ATIs; VBL is shown as positive control. **B.** Examples of MT-inhibitory phenotypes induced by ATIs 10 and ATI 57, followed in time after drug removal. MTs are visualized by FITC-conjugated anti-alpha-tubulin antibody (green) and chromosomes by DAPI staining of the DNA (blue). *Bar, 10* µm.

(*Continued*)

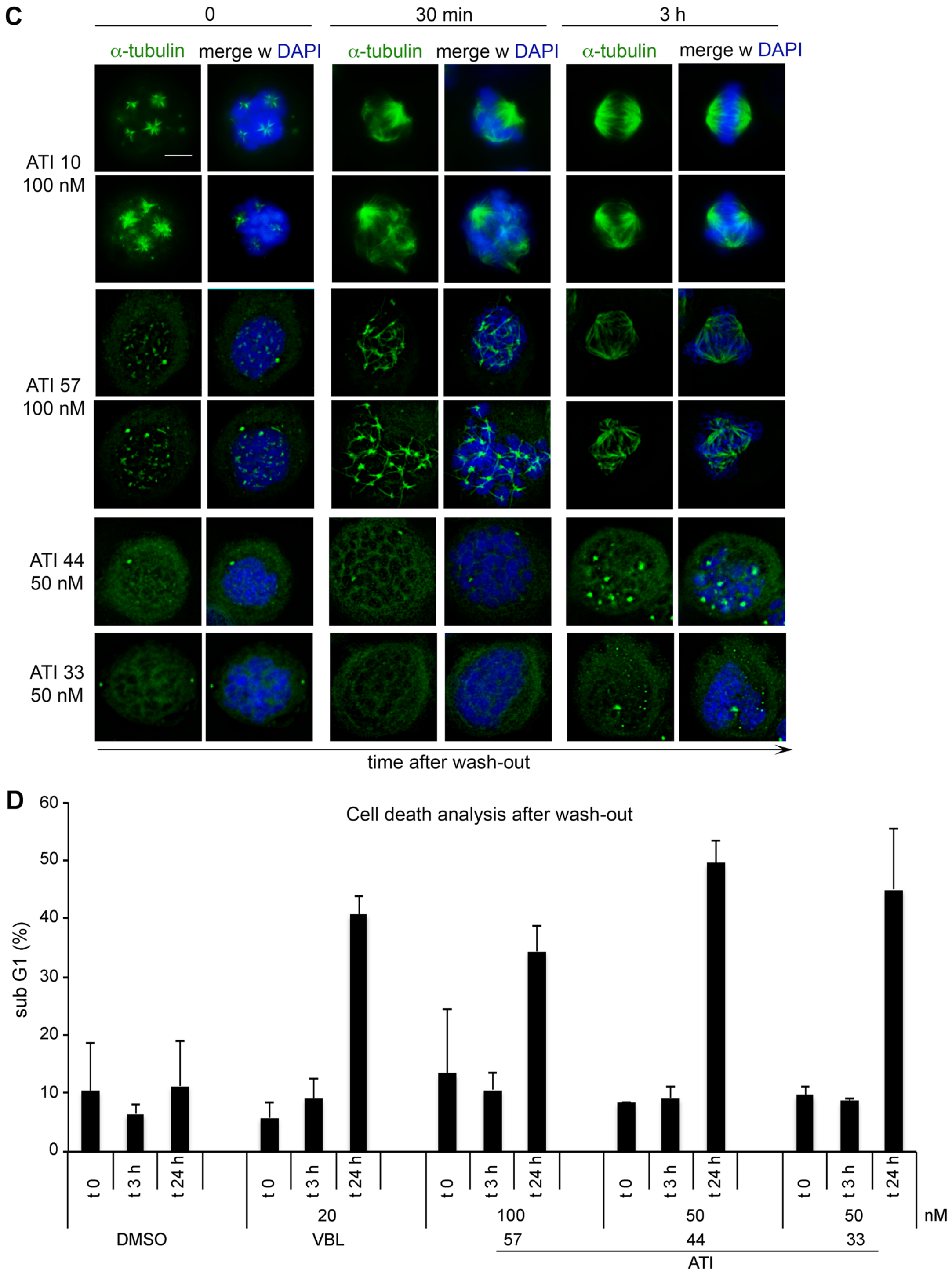


**Supplementary Figure 1 (*Continued*): C.** Examples of MT-inhibitory phenotypes recovering after drug removal, visualized in more detail for the indicated ATIs. *Bar*, 5 µm. **D.** Induction of cell death, visualized as % of sub-G1, after 24 hours of treatment (t0) or 3/24 hours after drug removal, with the indicated ATIs. For all assays, means and SD values were calculated from at least two independent experiments.

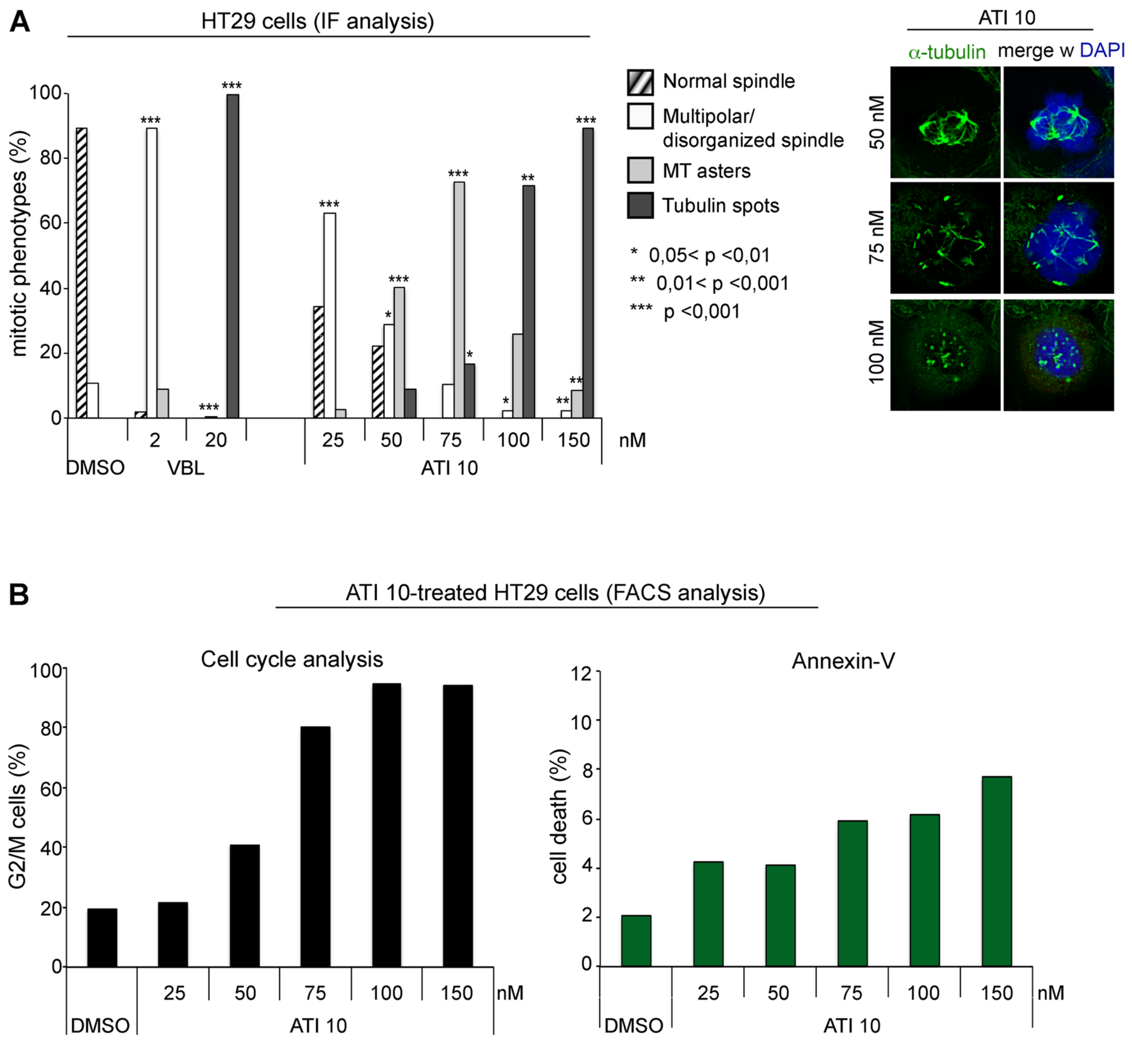


**Supplementary Figure 2: Dose-dependent induction of cell cycle arrest, cell death and MT- inhibitory phenotypes by ATI 10 in HT29 cultures. A.** Immunofluorescence analysis of mitotic figures: frequency of increasingly severe MT-inhibitory phenotypes (histograms). At least 100 mitotic cells were counted per ATI concentration (30 in DMSO, 400 in 20 nM VBL), $X^2$ test *p* value are indicated. **B.** FACS analysis of PI-stained (left panel) and Annexin V-FITC- stained HT29 after 24–hours- treatment with increasing concentrations of ATI 33.

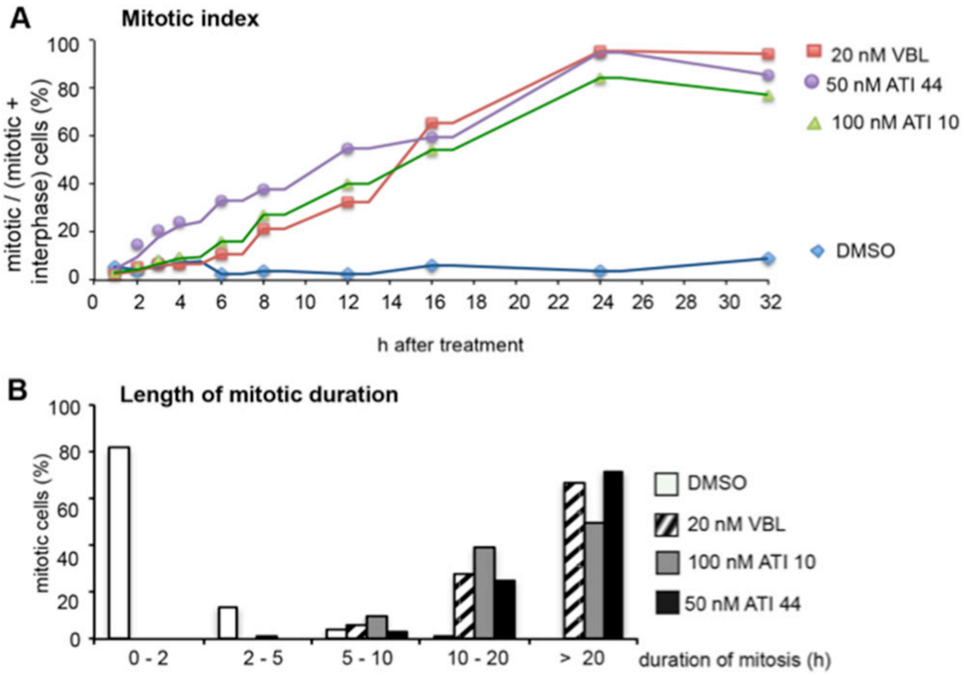


**Supplementary Figure 3: Timing of mitotic entry and mitotic arrest in HeLa cells treated with anti-mitotic drugs from the time of drug administration.** **A.** The graph represents the mitotic index in HeLa cultures exposed to VBL (20 nM), ATI 44 (50 nM), ATI 10 (100 nM) or DMSO for control, recorded from time 0 (from the moment of drug administration) over the next 32 hours. **B.** Mitotic cells from samples treated as for A are grouped according to the length of time that they spend in mitosis. The histograms represent the distribution of mitotic cells (in %) in the indicated time intervals. While most control cells accomplish mitosis in less than 2 hours, cells treated with antimitotic drugs induce durable mitotic arrest. Note that ATI 10 induces a slightly lower fraction of stably arrested cells (>20 hour-long arrest) compared to ATI 44 and VBL.
